# Unraveling Complexities: Rhabdomyolysis, Acute Renal Injury, and Compartment Syndrome Following a Wasp’s Sting

**DOI:** 10.7759/cureus.63938

**Published:** 2024-07-06

**Authors:** Manoj Kumar Kurmana, Maniram Kumhar, Ravindra Kumar Tiwari, Harsh Tak

**Affiliations:** 1 Internal Medicine, Jawaharlal Nehru Medical College Ajmer, Ajmer, IND

**Keywords:** intravenous hydration, insect bite complications, anaphylaxis, toxic systemic reactions, multiple wasp stings, myoglobinuria, wasp sting, compartment syndrome, acute renal injury, rhabdomyolysis

## Abstract

This study delves into the rare occurrence of rhabdomyolysis induced by wasp stings, emphasizing its toxic systemic repercussions. Drawing parallels with documented instances of insect bites worldwide, including those by honey bees and Africanized bees, the research explores the correlation between multiple wasp stings and acute renal failure associated with rhabdomyolysis. The venom’s active components, such as amines, kinins, and histamine-releasing peptides, underpin toxic systemic reactions, leading to hemolysis, coagulopathy, and severe cytotoxicity-induced acute renal failure. Noteworthy is the emergence of blackish necroses at the sting site, suggesting intense cytotoxicity. The study also highlights skin necrosis as a prognostic indicator for toxic systemic reactions. The presented case manifests an anaphylaxis-like reaction, revealing insights into toxic responses devoid of IgE-mediated allergic reactions. Timely intervention, encompassing hydration, transfusion, and dialytic support, proves imperative in scenarios involving multiple wasp stings, offering successful outcomes documented through plasma exchange in severe cases. This research prompts considerations beyond anaphylaxis, urging exploration of severe toxic systemic reactions in the context of multiple wasp stings.

## Introduction

Rhabdomyolysis is an acute, potentially fatal clinical syndrome [1] characterized by the breakdown of striated muscle tissue and the release of intracellular components into the systemic circulation. This condition often results in myoglobinemia and myoglobinuria, which are indicative of muscle damage and the release of myoglobin into the blood and urine. The release of other intracellular substances, such as calcium and potassium, can lead to significant systemic effects. Among the complications associated with rhabdomyolysis, prerenal azotemia and the nephrotoxicity of free myoglobin can precipitate acute kidney injury (AKI), further exacerbating metabolic abnormalities. Severe cases can result in fatal outcomes due to arrhythmias caused by hyperkalemia and hypocalcemia [2].

The main causes of rhabdomyolysis include muscle crush injuries, toxins, ischemia, metabolic disorders, and drugs [3]. While cases resulting from these causes are more common, rare instances have been reported following insect stings. Damage to skeletal muscle may result from physical harm to muscle cells or from interference with their blood supply [3].

Early identification and management of rhabdomyolysis are critical to preventing complications such as AKI and compartment syndrome. Diagnostic evaluation typically includes measuring serum creatine kinase (CK) levels, which can be markedly elevated, often exceeding five times the upper limit of normal. Myoglobinuria detected through routine urine testing, accompanied by proteinuria, further supports the diagnosis. Elevated markers of muscle injury, such as aldolase, aminotransferases, and lactate dehydrogenase, are also common findings. It is crucial to distinguish these enzyme elevations from liver involvement, as gamma-glutamyl transferase (GGT) and bilirubin are not present in muscle tissue, thereby guiding appropriate diagnostic and therapeutic approaches [4].

In this case report, we detail the clinical presentation, diagnostic evaluation, and management of a patient who developed rhabdomyolysis after a wasp sting, a rare but significant presentation. This report aims to contribute to the understanding of this uncommon complication and underscore the importance of early recognition and comprehensive management in preventing severe outcomes.

## Case presentation

A 20-year-old previously healthy male sought medical attention after being stung by a wasp. Initially, he experienced localized swelling and muscle aches at the sting site, which raised concerns about an allergic reaction, specifically anaphylaxis. Despite receiving standard treatments such as IV antihistamines and steroids, his symptoms persisted and worsened over time.

The patient's condition evolved to include increased swelling, persistent muscle aches, weakness, and a decline in urine output accompanied by dark-colored urine, suggestive of a more serious underlying condition. This progression prompted further investigation into potential complications, particularly rhabdomyolysis, a condition characterized by the breakdown of skeletal muscle tissue.

**Figure 1:**
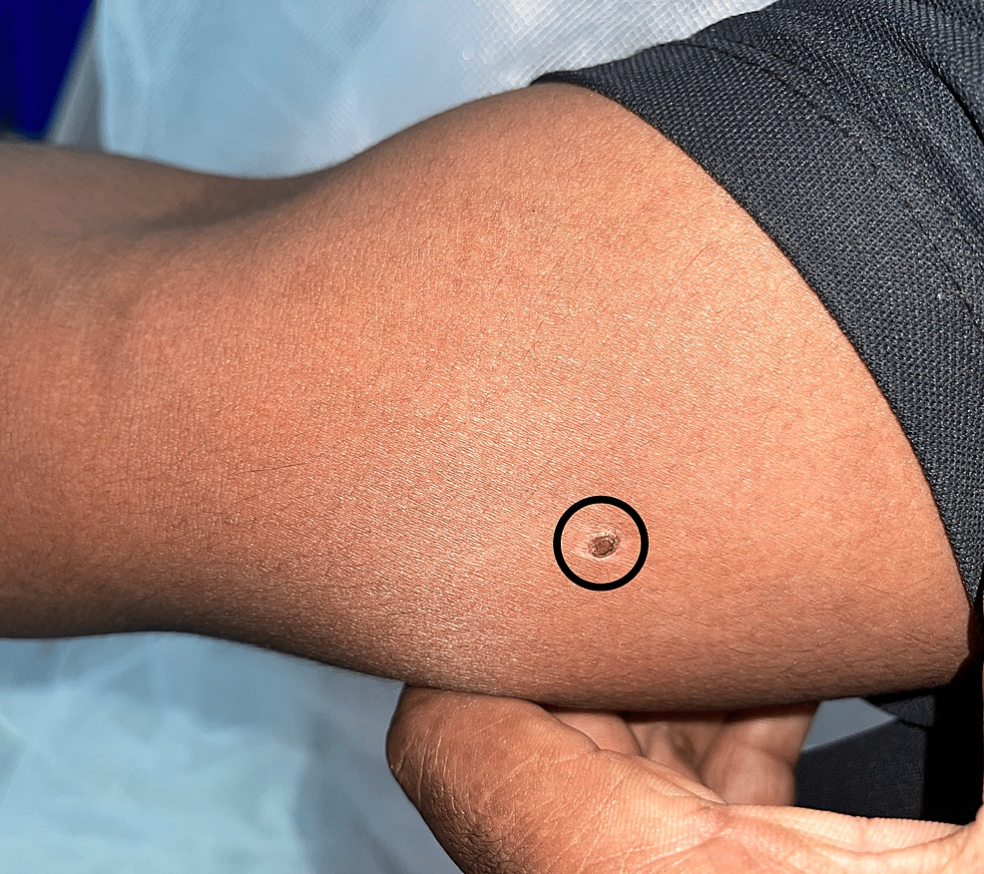
Image showing healed sting bite wound.

Upon physical examination, the patient's vital signs were stable, but there was noticeable swelling, erythema, and tenderness at the sting site (Figure 1). Laboratory tests (Table 1) revealed significantly elevated levels of creatine kinase with N-acetyl cysteine (CKNAC) and the presence of myoglobin in the urine, confirming the diagnosis of rhabdomyolysis. Imaging studies, including ultrasound, further supported the diagnosis by detecting muscle edema, while Doppler studies ruled out the possibility of deep vein thrombosis contributing to the patient's symptoms.

**Table 1:** Laboratory serum values during admission and discharge. SGOT: serum glutamic oxaloacetic transaminase, SGPT: serum glutamic pyruvic transaminase, AST: aspartate aminotransferase, ALT: alanine aminotransferase, CKNAC: creatine kinase with N-acetyl cysteine. Reference range cite: ABIM Laboratory Test Reference Ranges - January 2024. American Board of Internal Medicine [5].

Investigations	On admission (units)	On discharge (units)	Reference range
Sodium (Na)	140 mEq/L	133 mEq/L	136–145 mEq/L
Potassium (K)	4.7 mEq/L	3.9 mEq/L	3.5–5.0 mEq/L
Chloride (Cl)	95 mEq/L	96 mEq/L	98–106 mEq/L
Glucose (fasting)	75 mg/dL	92 mg/dL	70–99 mg/dL
CKNAC	1363 U/L	132 U/L	55–170 U/L (male)
SGOT (AST)	1500 U/L	645 U/L	10–40 U/L
SGPT (ALT)	500 U/L	234 U/L	10–40 U/L
Bilirubin total	0.5 mg/dL	0.8 mg/dL	0.3–1.0 mg/dL
Creatinine (Cr)	0.6 mg/dL	0.8 mg/dL	0.70–1.30 mg/dL
Urea	65 mg/dL	55.4 mg/ dL	8–20 mg/dL

There, we started treatment promptly to address the underlying condition and prevent further complications. This included intravenous administration of crystalloids to support renal function and promote urine output, antihistamines to manage the allergic reaction, and steroids to reduce inflammation. Additionally, the patient received a tetanus toxoid vaccination as a preventive measure against tetanus infection.

Close monitoring for potential complications, particularly compartment syndrome, was essential throughout the treatment process. Compartment syndrome occurs when increased pressure within a muscle compartment compromises blood flow and can lead to tissue damage. By closely monitoring the patient's symptoms and response to treatment, healthcare providers were able to intervene promptly and effectively manage any complications that arose.

Over the course, the patient showed signs of improvement, with an increase in urine output and a decrease in CK levels, indicating a positive response to treatment. This case serves as a reminder of the importance of prompt medical attention for insect stings and the critical role of healthcare providers in recognizing and managing potential complications, such as rhabdomyolysis, to ensure optimal patient outcomes.

## Discussion

Rhabdomyolysis is characterized by the breakdown of muscle tissue, resulting in the release of intracellular muscle components into the bloodstream. Clinically, it presents with symptoms such as muscle pain, weakness, and the presence of dark-colored urine due to myoglobin excretion. Diagnosis typically relies on elevated CK levels, often exceeding five times the upper limit of normal, ranging approximately from 1500 to over 100,000 units/L. Detection of myoglobinuria through routine urine testing, accompanied by proteinuria, aids in confirmation. Additionally, markers of muscle injury, such as aldolase, aminotransferases, and lactate dehydrogenase, are commonly elevated. Although these enzyme elevations may initially suggest liver involvement, the absence of GGT and bilirubin in muscle emphasizes the distinction. Elevated CK alongside normal serum GGT or bilirubin levels directs diagnostic efforts toward muscle injury, avoiding unnecessary investigations related to liver function. Serum aminotransferases, while increased, lack specificity and can indicate tissue damage from either muscle or liver sources. In one study, AST was elevated in 93% and ALT in 75% of rhabdomyolysis cases with CK greater than or equal to 1000 units/L [6,7]. Only in one instance was the ALT greater than the AST, although the AST declines faster than the ALT as the rhabdomyolysis resolves, such that the two may equalize after a few days, as seen in our case. Rhabdomyolysis induced by wasp stings is an exceptionally uncommon occurrence, attributed to the poisonous impact of wasp venom without provoking an allergic response [7]. Mejía-Vélez [8] detailed 43 incidents of acute renal failure resulting from numerous stings by the Africanized bee, demonstrating a correlation with rhabdomyolysis. While a solitary sting can incite IgE-mediated anaphylaxis, mass stings can elicit systemic responses characterized by toxin-mediated cellular damage.

Analogous conditions have been documented in global instances of insect bites, including those inflicted by honey bees, Africanized bees, and Hymenoptera [8-10]. The venom of wasps comprises active amines, including serotonin and histamine, as well as wasp kinins and peptides that release histamine [11]. These components underlie toxic systemic reactions, encompassing hemolysis, coagulopathy, rhabdomyolysis, and severe cytotoxicity-induced acute renal failure [12]. In some rare cases, the emergence of blackish necroses at the sting site after one month suggests intense cytotoxicity attributable to the wasp venom. Japan has reported 16 instances of rhabdomyolysis associated with wasp stings, 15 of which manifested skin necrosis [12,13]. Youichi et al. have highlighted skin necrosis as an ominous prognostic indicator for toxic systemic reactions post-wasp stings [14]. Additionally, our case exhibited an anaphylaxis-like reaction, purportedly triggered by a toxic response devoid of IgE-mediated allergic reactions, supported by the normal range of wasp-specific IgE and the absence of wheal and respiratory symptoms. Timely intervention assumes paramount importance in scenarios involving multiple wasp stings, necessitating the identification of complicating toxin-related injuries. While epinephrine and steroids are advocated for anaphylaxis, comprehensive hydration becomes imperative in cases of toxin-related multi-system injuries to forestall renal impairment stemming from rhabdomyolysis alongside these agents [15]. Hemolysis and rhabdomyolysis stand as pivotal contributors to acute renal failure; hence, their scrutiny becomes imperative in instances of multiple wasp stings [15]. Given the profound impact of acute renal failure on the prognosis of wasp stings, expeditious transfusion and dialytic support have become imperative [16]. Successful outcomes have been documented with plasma exchange in severe cases [17]. It is imperative to contemplate not solely anaphylaxis but also the potentiality of severe toxic systemic reactions in scenarios involving multiple wasp stings. In the context of managing patients with rhabdomyolysis, it is essential to assess potential underlying causes, especially when the cause is not evident from the patient’s history, physical examination, or initial lab results. This evaluation should include consideration of inherited factors or susceptibilities to rhabdomyolysis, such as metabolic myopathies or muscular dystrophies. Additionally, the primary treatment goals involve preventing acute kidney injury (AKI) and related metabolic complications, along with managing acute compartment syndrome [18].

## Conclusions

It is imperative for individuals who have been stung by bees or wasps to seek prompt medical attention, even if they initially appear unaffected. However, unusual reactions happen mostly after massive stings (like Africanized bees or yellow jackets), but in this case, the reaction follows a solitary wasp sting. Primary care physicians should maintain a high level of awareness regarding potential complications, including anaphylaxis and damage to vital organs such as the kidneys, liver, and muscles. This case serves as a reminder of the critical importance of early recognition and treatment of conditions such as rhabdomyolysis with compartment syndrome and acute kidney injury. Timely intervention is essential to prevent the development of chronic kidney disease and reduce mortality rates among affected patients.
